# The Effect of Maternal Diet with Fish Oil on Oxidative Stress and Inflammatory Response in Sow and New-Born Piglets

**DOI:** 10.1155/2019/6765803

**Published:** 2019-06-02

**Authors:** W. L. Luo, Z. Luo, X. Xu, S. Zhao, S. H. Li, T. Sho, J. Yao, J. Zhang, W. N. Xu, J. X. Xu

**Affiliations:** ^1^School of Agriculture and Biology, Shanghai Jiao Tong University, Shanghai Key Laboratory of Veterinary Biotechnology, Shanghai 200240, China; ^2^Division of Animal and Nutritional Sciences, West Virginia University, Morgantown, WV 26506, USA

## Abstract

Pregnancy is an oxidative stress and immune challenge for the mother. Fish oil is rich in EPA and DHA, which can partly inhibit aspects of inflammation and restore antioxidant capacity. In the present study, we investigated the effect of maternal diet with fish oil during the late gestation period on oxidative stress and inflammatory response in sows and their progenies. Twelve second-parity sows were allocated equally into two groups. Sows were fed either the soybean oil diet (SD) or soybean oil+fish oil diet (FD) during the gestation period. The plasma of sows, cord blood, and new-born piglets were collected. Full-term placentas and livers of new-born piglets were also sampled. The activities of glutathione peroxidase (GSH-Px) and total superoxide dismutase (T-SOD) in the plasma of sows on farrowing day were higher, and the concentrations of interleukin-6 (IL-6) and prostaglandin-endoperoxide synthase 2 (PGE2) in the plasma of sows on farrowing day and interleukin-1*β* (IL-1*β*) in the plasma of cord blood were lower in the FD group than those in the SD group (*P* < 0.05). The FD downregulated the expression of SOD, IL-1*β*, IL-6, and transforming growth factor-*β*-activated kinase 1 binding protein 1 (TAB1) mRNA but upregulated the expression of lipoxygenase enzyme 5 (ALOX5) and interleukin-10 (IL-10) mRNA in placentas (*P* < 0.05). The FD downregulated the protein expression level of p-JNK/JNK in placentas (*P* < 0.05). In the livers of new-born piglets, the FD upregulated the expression of ALOX5 (*P* < 0.05) and G-protein-coupled receptor 120 (GPR120) (*P* < 0.05) mRNA. Our results suggest that the maternal diet with fish oil might alleviate oxidative stress in sows on farrowing day and modulate inflammatory response in full-term placentas by inhibiting the JNK signal pathway. Moreover, the maternal diet with fish oil might partly regulate the neonatal immune response of their progenies.

## 1. Introduction

Pregnancy is often described as a state of systemic inflammation [1]. During the late gestation period, the rapid development of the foetus leads to increased metabolic burdens on pregnant women or dams, causing elevated systemic oxidative stress [2, 3]. The oxidative stress exacerbates the maternal systemic inflammatory response to pregnancy and aberrant cytokine expression and vice versa [4]. Moreover, labour-induced injuries of the birth canal and uterus are regarded as another important reason for the aggravated oxidative stress and inflammatory response during the perinatal period [5, 6]. Previous reports showed that mothers who experienced oxidative stress and inflammatory responses during late gestation may give rise to paediatric illness or gynaecological problems, such as hypoxia-ischemia, perinatal asphyxia, preterm birth, preeclampsia (PE), miscarriage and gestational diabetes mellitus [7–9].

Fish oil is used as a source of long-chain *n* − 3 polyunsaturated fatty acids (*n* − 3 LC-PUFA) by providing eicosapentaenoic acid (EPA; 20 : 5*n* − 3) and docosahexaenoic acid (DHA; 20 : 6*n* − 3) [10] and has been reported to restore antioxidant capacity because of the high content of EPA and DHA [11]. Moreover, the anti-inflammatory properties of DHA and EPA are well documented in vitro or in experimental animals, whereby they inhibit the production of proinflammatory eicosanoid (e.g., Prostaglandin E2, PGE2) and proinflammatory cytokine (e.g., IL-1*β*, IL-6, and TNF-*α*) and inhibit the activity of NF-*κ*B and MAPK (ERK, JNK, and p38) [12–14]; they stimulate the production of specialized proresolving lipid mediators (SPMs) and anti-inflammatory cytokines (e.g., IL-10) [15, 16]. Sow placenta is an integral component of inflammatory response during the gestation period as it actively produces a variety of cytokines and immunomodulatory hormones and is sensitive to the systematic oxidative status. Labour is a powerful inducer of placental oxidative stress and inflammatory cytokines [6]. Maternal dietary *n* − 3 LC-PUFA supplementation has been recognized as an important factor to reduce placental oxidative stress and enhance placental and foetal growth in rats [17]. Inconsistent with the report on rats, maternal diet with fish oil decreased inflammatory response in sows and their offspring but increased the susceptibility to oxidative stress in sows and piglets [18–20]. Furthermore, there are no reports about the effect of maternal diet with *n* − 3 LC-PUFA on oxidative stress and inflammation response in new-born offspring before suckling. In placental mammals, the umbilical vein supplies the foetus with oxygenated, nutrient-rich blood from the placenta by supplying blood to the liver of the foetus via the hepatic portal vein [21]. Therefore, the placenta, cord blood, and liver of neonatal pigs play an important role in the cross talk of nutrients, oxidative stress, and inflammation between the sows and their offspring. Previous reports showed that the fatty acid composition in the placenta, cord blood, and livers of new-born piglets was changed by the maternal diet with *n* − 3 PUFA [22, 23]. As mentioned above, we hypothesized that the maternal diet with fish oil might inhibit oxidative stress and inflammatory response in sow, cord blood, and new-born offspring, and the placenta and the liver of the foetus might play an important role in this process.

In the present study, we investigated the effect of maternal diet with fish oil on the oxidative stress status and inflammatory response in sows and new-born piglets on farrowing day. We analysed the NF-*κ*B and MAPK signal pathways in the placenta and livers of piglets and evaluated the possible mechanism for the *n* − 3 LC-PUFA to regulate oxidative stress status and inflammatory response.

## 2. Materials and Methods

All experimental protocols were approved by the Animal Care and Use Committee of the Shanghai Jiao Tong University. The study took place at the experimental study farm of a feeding company (Shanghai Xinnong Feed Co. Ltd., Shanghai, China) during May and June 2016.

### 2.1. Experimental Design and Animal Management

Twelve second-parity sows (hybrid Topigs 20 breed sows, Dutch Landrace×Great York) and their piglets ((Dutch Landrace×Great York)×Duroc) were used in the experiment. Sows were inseminated with semen from Duroc boars. On day 85 of gestation, sows were equally divided into two groups, with six replicates per group and one sow per replicate. The back fat thickness of sows was measured at day 84 of gestation, and 12 sows which had similar back fat thickness (soybean oil group: 15.50 ± 0.61 vs. fish oil group: 14.83 ± 0.79 mm; *P* = 0.52) were selected for our study. The back fat thickness was measured at the level of the last rib on each side and 65 mm from the midline by using a digital back fat indicator (BQT-521, Renco Lean-Meater, USA).

From mating until day 84 of gestation, the sows were kept individually in crates and were fed a standard gestation diet (Shanghai Xinnong Feed Co. Ltd., Shanghai, China). During the trial period, all 12 sows were divided into two dietary treatment groups: the first group was fed the soybean oil maternal diet (SD), and the second group was fed the fish oil maternal diet (FD) during the gestation period. In each group, sows were fed gestation diet 1 and gestation diet 2 according to the day of gestation. Diets were formulated according to the sow's nutrient requirements from the National Research Council (NRC, 2012) [24]. Soybean oil and fish oil were analysed for fatty acid composition (Supplementary [Supplementary-material supplementary-material-1]). For gestation diet 1, the SD was composed of 3% soybean oil to make the *n* − 6 : *n* − 3 PUFA ratio 8.8 : 1, while the FD was composed of 0.5% soybean oil+2.5% fish oil to make the *n* − 6 : *n* − 3 PUFA ratio 1.6 : 1. For gestation diet 2, the SD was composed of 3.5% soybean oil to make the *n* − 6 : *n* − 3 PUFA ratio 10 : 1, while the FD was composed of 0.7% soybean oil+2.8% fish oil to make the *n* − 6 : *n* − 3 PUFA ratio 2 : 1. Diet formulations and fatty acid composition for the diets are shown in Tables 1 and 2, respectively. The fatty acid composition in diets was determined as described by Raes et al. [25]. All diets were mash feed and were stored in vacuum dark storage bags per 20 kg and kept in a warehouse with 24-28°C constant temperature before using.

From day 85 to day 109 of gestation, all pregnant sows were housed individually in gestation crates (2.1 × 0.65 m) and were fed gestation diet 1. Gestation diet 1 was supplied twice a day (06:00 and 13:00) and supplied at 3.0 kg/day (the 3.0 kg/day diet was limited to sows during the late gestation period, so sows can eat up to 3.0 kg/day). On day 110 of gestation, all pregnant sows were transferred to farrowing units (2.23 × 2.2 m). From day 110 of gestation to farrowing day, gestation diet 2 was supplied three times a day (06:00, 13:00, and 18:00) beginning at 2.5 kg/day, then feed allowance was reduced by 0.5 kg/day until farrowing day when no diet was provided. The room temperature of the gestation and farrowing units was approximately 24–28°C. Sows had free access to water during the entire experiment.

### 2.2. Farrowing and Piglets

Parturitions were not induced and were attended. At parturition, the duration of gestation was recorded immediately. Twelve new-born piglets (3 males and females in the SD group and the FD group) were selected in the study. One piglet per litter (body weight close to the average weight of the litter) was selected at birth (before suckling), and the corresponding placentas of selected piglets were collected. The average body weight of new-born piglets is similar between the SD group and the FD group (SD: 1.54 ± 0.14*vs.* FD: 1.41 ± 0.05 kg; *P* = 0.42). The internal organs (intestine, liver, kidney, spleen, heart, and pancreas) of new-born pigs were measured to calculate the relative organ weight and length to birth weight. After expulsion, placentas were collected and their weights were recorded. The ratio of piglet weight : placental weight was used as a measure of placental efficiency.

### 2.3. Blood and Tissue Sample Collection

#### 2.3.1. Blood Samples

Blood samples from the sows were collected from the jugular vein on day 84 of gestation (G84d) and on farrowing day (Fd). Umbilical cord blood was collected by squeezing from the retracted side of the umbilical cord. Each piglet was anaesthetized with an intramuscular neck injection of pentobarbital sodium (35 mg/kg BW), and a blood sample was then collected from the vena jugulars. All blood samples were kept in heparinized tubes and centrifuged at 2550 ×*g* for 10 min at 4°C. The supernatant fraction was divided and stored at −20°C for subsequent analysis.

#### 2.3.2. Placenta Samples

Each foetus and the corresponding placenta were carefully marked. Sows were closely supervised throughout farrowing and each new-born piglet was matched to its placenta using the umbilical tagging procedure described by Wilson et al. [26]. Placenta samples around the central cord region of the placenta were obtained immediately after farrowing and collected into 5 mL freezing tubes, frozen in liquid nitrogen, and then stored at −80°C for further analysis.

#### 2.3.3. Liver Samples of Piglets

The posterior half of liver samples were obtained immediately after slaughtering. The tissues were washed in physiological saline, collected into 5 mL freezing tubes, frozen in liquid N_2_, and then stored at −80°C.

### 2.4. Analysis of Oxidant and Antioxidant Contents

The analysis of malondialdehyde (MDA), total superoxide dismutase (T-SOD), glutathione peroxidase (GSH-Px), and total antioxidant capacity (T-AOC) in plasma was determined according to the manufacturer's instructions (Nanjing Jiancheng Bioengineering Institute, Nanjing, China). Briefly, the content of MDA was measured with 2-thiobarbituric acid at 95°C to generate a coloured product with an absorbance at 532 nm. T-SOD activity was analysed based on the mechanism that superoxide inhibits the nitro blue tetrazolium reduction. One unit of SOD activity is defined as the amount that reduces the absorbance at 550 nm by 50% in 1 mL of plasma. GSH-Px activity was assayed by measuring the reduction of glutathione per min at 412 nm after the subtraction of the nonenzymatic reaction. The T-AOC in plasmas was assayed by the reduction of Fe^3+^-tripyridyltriazine to Fe^2+^-tripyridyltriazine and expressed as U/mL according to the method of Hu et al. [27]. All absorbance levels were determined with a microplate reader (Synergy 2, BioTek Instruments Inc., USA).

### 2.5. Analysis of Cytokines in Plasma

The concentrations of cytokines including interleukin-1*β* (IL-1*β*), interleukin-6 (IL-6), Tumor necrosis factor (TNF-*α*), interleukin-10 (IL-10), and prostaglandin E2 (PGE2) was determined using porcine-specific ELISA kits (Nanjing Jiancheng Bioengineering Institute, Nanjing, China) according to the manufacturer's instructions. Absorbance values were read in a 96-well plate reader (Synergy 2, BioTek Instruments Inc., USA) at 450 nm. A four-parameter logistic curve fit was generated using ELISA Calc software v0.1 (Complete-Software, Iowa City, IA). The concentrations of cytokines and PGE2 in the plasma was calculated by comparison with a standard curve.

### 2.6. Quantitative Real-Time PCR

Total RNA was isolated from placenta and liver samples using Total RNA Kit I (50) (Cat. No. R6834-01; Omega Bio-tek Inc., USA). RNA quality was verified by both agarose gel (1%) electrophoresis and spectrometry (A260/A280, Beckman DU-800; Beckman Coulter Inc.). One microgram of RNA was reverse transcribed using the PrimeScript™ RT Reagent Kit with the gDNA Eraser (Perfect Real Time) (RR047A, TaKaRa, Japan). Primers for all target genes are shown in Supplementary [Supplementary-material supplementary-material-1]. Quantitative real-time PCR (RT-qPCR) was used to determine the relative expression level of target genes using the one-step SYBR® Premix Ex Taq (TLi RNaseH Plus) (RR420A, TaKaRa, Japan). Briefly, the final volume of the reaction mixtures (20 *μ*L) contained 10 *μ*L of SYBR Premix Ex Taq (Tli RNaseH Plus), 0.8 *μ*L of the primer pair, 0.4 *μ*L of ROX Reference Dye II, 2 *μ*L of cDNA, and 6.8 *μ*L of sterile water. *β*-Actin was used as the endogenous control gene to normalize the expression of target genes. The relative quantification of gene amplification by RT-qPCR was performed using the value of the threshold cycle (*C*_t_). The comparative *C*_t_ value method using the formula 2^−ΔΔ*C*_t_^ was employed to quantify the expression levels of target genes relative to those of *β*-actin [28]:

(1) $$\begin{array}{c} 2^{-\Delta \Delta C_{\text{t}}}\left(\Delta \Delta C_{\text{t}}=\left(C_{\text{t}}\text{ target gene}–C_{\text{t}}\;\beta ‐\text{actin}\right)\text{ treatment}–\left(C_{\text{t}}\text{ target gene}–C_{\text{t}}\;\beta ‐\text{actin}\right)\text{control}\right). \end{array}$$

### 2.7. Western Blot Analysis

The proteins in the placenta and liver samples were extracted and mixed with a loading buffer as previously described by Luo et al. [29]. Forty micrograms of proteins was separated on 10% SDS-PAGE gels and electrotransferred to polyvinylidene difluoride (PVDF) membranes (0.45 *μ*m pore size, IPVH00010, Millipore, MA). The membranes were blocked for 2 h with 5% (*w*/*v*) skimmed milk powder (Cat. No. D8340, Shanghai Solarbio Science & Technology Co. Ltd., Shanghai, China) in Tris-buffered saline-Tween (TBS-T) (0.5 M NaCl (S7653, Sigma-Aldrich, Shanghai, China), 20 mM Tris (Amresco, Shanghai, China), pH 7.5, and 0.1% (*v*/*v*) Tween-20 (P7949, Sigma-Aldrich, Shanghai, China), then washed three times with TBS-T and incubated overnight at 4°C with primary antibodies following dilutions in 5% skimmed milk powder or BSA (Cat. No. 0218054950, ChromatoPur™, New Zealand). The primary antibodies were anti-JNK (1 : 200, sc-571, Santa Cruz Biotechnology Inc., USA), anti-p-JNK (1 : 500, orb10951, Biorbyt Ltd., UK), anti-p38*α* (1 : 2000, sc-535, Santa Cruz Biotechnology Inc., USA), anti-p-p38 (1 : 200, sc-7973, Santa Cruz Biotechnology Inc., USA), anti-ERK1/2 (1 : 1000, number 9102, Cell Signaling Technology, USA), anti-phospho-ERK1/2 (1 : 2000, number 4370, Cell Signaling Technology, USA), anti-I*κ*B*α* (1 : 1000, number 4814, Cell Signaling Technology, USA), and anti-p-I*κ*B*α* (1 : 1000, number 9246, Cell Signaling Technology, USA). After washing three times with TBS-T, the membranes were then incubated with goat anti-rabbit (1 : 10000, ab97051, Abcam, UK) or goat anti-mouse IgG-HRP (1 : 2000, sc-2005, Santa Cruz Biotechnology Inc., USA) antibodies for 2 h. Afterwards, blots were developed using Amersham™ ECL™ Prime Western Blotting Detection Reagent (Catalogue No. RPN2232, GE Healthcare UK Ltd., Little Chalfont). Image acquisition was performed on an enhanced chemiluminescence detection system (Tanon, Shanghai, China). ImageJ software was used to quantify the density of the specific protein bands.

### 2.8. Statistical Analyses

All variables were tested for normal distribution by the Shapiro-Wilk test. All data were normally distributed. An individual sow or piglet was the experimental unit for the indices. All data were analysed by using the procedure of the *t*-test (IBM SPSS Statistics 20). Results were expressed as means and the standard error of the mean (SEM). A *P* value less than 0.05 was considered to be statistically significant.

## 3. Results

### 3.1. Pregnancy Outcome and Organ Indices of Piglets

The duration of gestation was increased by the FD (*P* < 0.01), but the FD had no effect on the placenta weight and placental efficiency (*P* > 0.05) (Table 3).

The organ indices of new-born piglets were shown in Table 3. There were no differences in the relative intestinal length and pancreas weight of new-born piglets between the SD group and the FD group. The FD had no effect on the relative liver weight, brain weight, spleen weight, and kidney weight of the piglets.

### 3.2. Effect of Maternal Diet with Fish Oil on the Oxidative Stress Status in Sows and New-Born Piglets

**Figure 1:**
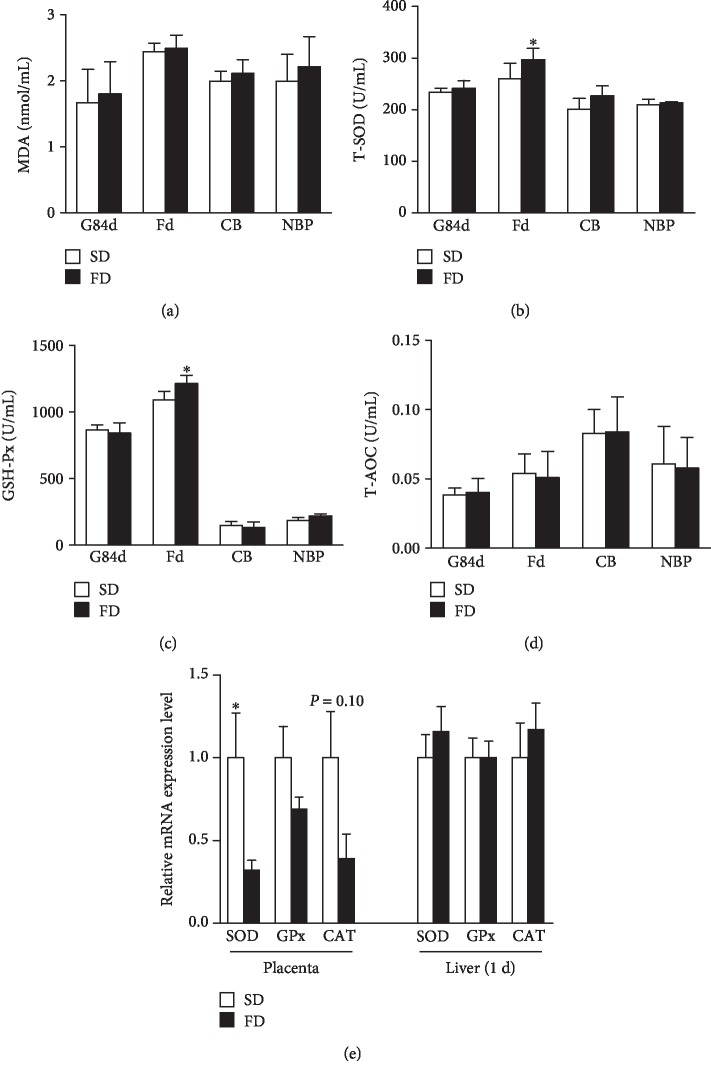
Effect of maternal diet with fish oil on the oxidative stress status in sows and new-born piglets. MDA concentration (a), T-SOD activity (b), GSH-Px activity (c), and T-AOC (d) in plasma collected from sows on the 84th day of gestation (G84d) and farrowing day (Fd), cord blood (CB), and new-born piglets (NBP) were determined. Relative expression levels of SOD, GPx, and CAT mRNA in placentas and livers of new-born piglets collected from sows fed different diets (e) were determined by real-time PCR. Values are means (*n* = 6), with their standard errors represented by vertical bars. ^∗^Mean values were significantly different between the two diet groups (*P* < 0.05). SD=soybean oil diet; FD=fish oil diet.

The FD had no effect on the MDA and T-AOC in sow plasma at farrowing day (*P* > 0.05) (Figures 1(a) and 1(c), respectively), while the activities of T-SOD and GSH-Px in the plasma of sows on farrowing day were higher in the FD group than in the SD group (*P* < 0.05) (Figures 1(b) and 1(c), respectively). Conversely, the FD significantly downregulated the relative expression of SOD mRNA in placentas (*P* < 0.05) (Figure 1(e)).

### 3.3. Effect of Maternal Diet with Fish Oil on the Concentrations of Cytokines in Plasma and mRNA Expression of Cytokines in Placentas and Livers of New-Born Piglets

**Figure 2:**
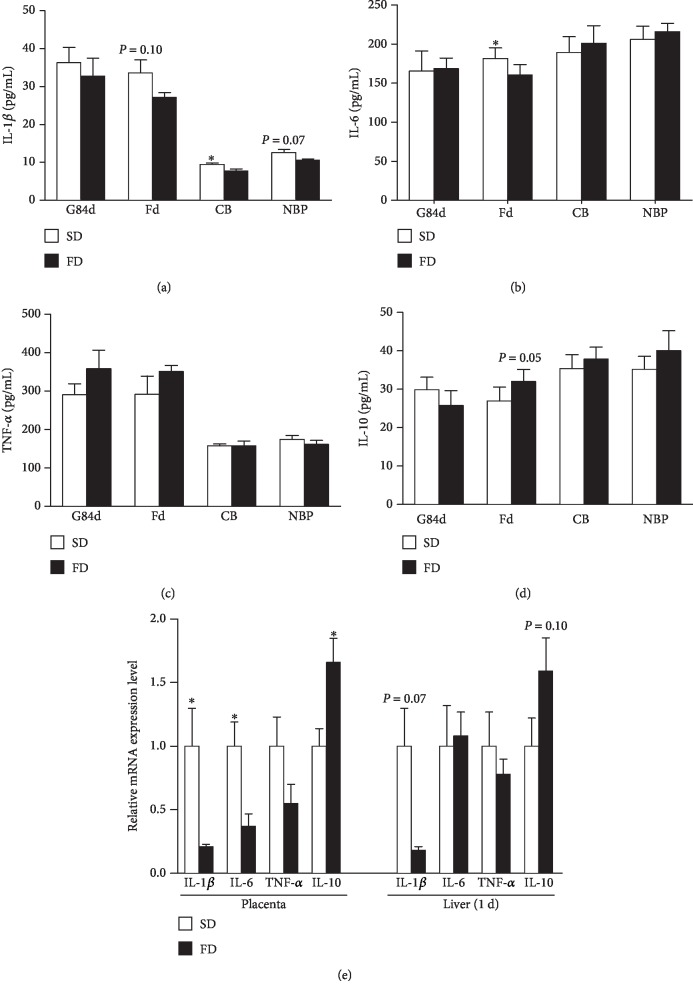
Effect of maternal diet with fish oil on the concentrations of cytokines in plasma and mRNA expression of cytokines in placentas and livers of new-born piglets. IL-1*β* concentration (a), IL-6 concentration (b), TNF-*α* concentration (c), and IL-10 concentration (d) in plasma collected from sows on the 84th day of gestation (G84d) and farrowing day (Fd), cord blood (CB), and new-born piglets (NBP) were determined. Relative expression levels of IL-1*β*, IL-6, and TNF-*α* and IL-10 mRNA in placentas and livers of new-born piglets collected from sows fed different diets (e) were determined by the real-time PCR method. Values are means (*n* = 6), with their standard errors represented by vertical bars. ^∗^Mean values were significantly different between the two diet groups (*P* < 0.05). SD=soybean oil diet; FD=fish oil diet.

The FD decreased the concentration of IL-1*β* in the plasma of cord blood (*P* < 0.05), and it had a tendency to decrease IL-1*β* in the plasma of new-born piglets (*P* = 0.07) (Figure 1(a)). The concentration of IL-6 in the plasma of sows on farrowing day was significantly decreased in the FD group (*P* < 0.05) (Figure 2(b)). Conversely, the FD had a tendency to increase IL-10 (*P* = 0.05, Figure 2(d)) in the plasma of sows on farrowing day. In addition, the concentrations of TNF-*α* in the plasma of sows on farrowing day, cord blood, and new-born piglets were not different between the groups (Figure 2(c)).

The relative expression of IL-1*β* and IL-6 mRNA in placentas of the FD group was lower than those of the SD group (*P* < 0.05), while the relative expression of IL-10 mRNA in placentas of the FD group was higher than that of the SD group (*P* < 0.05) (Figure 2(e)). In the livers of new-born piglets, the FD had a tendency to downregulate the expression of IL-1*β* mRNA (*P* = 0.07) but upregulate the expression of IL-10 mRNA (*P* = 0.08) (Figure 2(e)).

### 3.4. Effect of Maternal Diet with Fish Oil on Anti-Inflammatory Parameters in Placentas and Livers of New-Born Piglets

**Figure 3:**
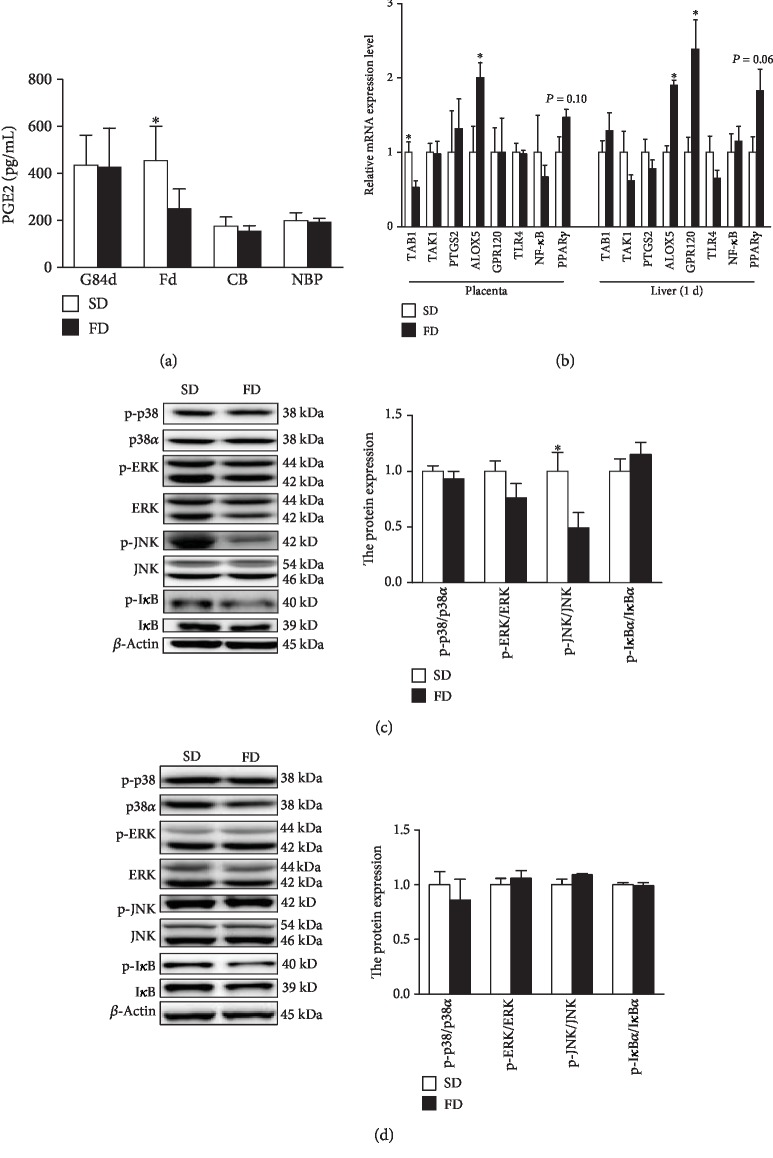
Effect of maternal diet with fish oil on anti-inflammatory parameters in placentas and livers of piglets. PGE2 concentrations (a) in plasma collected from sows on the 84th day of gestation (G84d) and farrowing day (Fd), cord blood (CB), and new-born piglets (NBP) were determined by the ELISA method. Relative expression levels of TAB1, TAK1, PTGS2, Alox5, GPR120, TLR4, NF-*κ*B, and PPAR*γ* mRNA in the placentas and livers of new-born piglets collected from sows fed different diets (b) were determined by real-time PCR. The protein expression of JNK, p-JNK, p38*α*, p-p38, ERK1/2, phospho-ERK1/2, I*κ*B*α*, and p-I*κ*B*α* in the placentas (c) and livers (d) of new-born piglets collected from sows fed different diets was determined by Western blot analysis. The values are calculated as the ratios of phosphorylation levels (p-JNK, p-p38, p-ERK1/2, and p-I*κ*B) to the total levels of JNK, p38, ERK1/2, and I*κ*B. Values are means (*n* = 6), with their standard errors represented by vertical bars. ^∗^Mean values were significantly different between the two diet groups (*P* < 0.05). SD=soybean oil diet; FD=fish oil diet. Note: p38 (p38*α* (C20), SC-535, Santa Cruz Biotechnology Inc.) is recommended for the detection of p38*α* as the total protein, while p-p38 (p-p38 (D-8) SC7379, Santa Cruz Biotechnology Inc.) is recommended for the detection of p38*α*, p38*β*, and p38*γ* correspondingly.

The concentration of PGE2 in the plasma of sows on farrowing day was significantly decreased in the FD group (*P* < 0.05) (Figure 3(a)). There is no diet effect on the relative expression of PTGS2 mRNA in placentas and livers of new-born piglets, while the relative expression of lipoxygenase enzyme 5 (ALOX5) mRNA in placentas of the FD group was higher than that of the SD group (*P* < 0.05) (Figure 3(b)). There was a tendency of upregulation of peroxisome proliferator activated receptor gamma (PPAR*γ*) mRNA expression in the livers of new-born piglets of the FD group (*P* = 0.06) (Figure 3(b)). The relative expression of transforming growth factor-*β*-activated kinase 1 binding protein 1 (TAB1) mRNA in the placenta was lower in the FD group than that in the SD group. Moreover, the FD significantly upregulated the relative expression of the ALOX5 and G-protein-coupled receptor 120 (GPR120) mRNA in the livers of new-born piglets (*P* < 0.05) (Figure 3(b)).

The diet of sows with fish oil significantly downregulated the p-JNK/JNK protein in placentas (*P* < 0.05), but had no effect on the p-JNK/JNK protein in livers of new-born piglets (Figure 3(c)). In addition, the expression of p-p38/p38*α*, p-ERK/ERK, and p-I*κ*B*α*/I*κ*B*α* proteins in placentas (Figure 3(c)) and livers (Figure 3(d)) of new-born piglets did not show any differences between the two groups.

## 4. Discussion

The primary objective of this study was to investigate the effect of maternal diet with fish oil on the oxidative stress status and inflammatory response in sows and new-born piglets on farrowing day and then to evaluate the possible mechanism for the *n* − 3 LC-PUFA to regulate oxidative stress status and inflammatory response.

The fat and protein accretion in the foetus accelerates after day 69 of gestation, but increasing the provision of dietary fat or energy to pregnant sows on day 70 of gestation may not be an ideal means for increasing the sow's reproductive performance because excessive maternal fat supply during the gestation period decreases voluntary feed intake during lactation [30, 31] and decreases sow longevity [32]. Therefore, the 85th day of pregnancy has been chosen for the start of the experimental diet. In our study, the duration of gestation was significantly influenced by the gestation diet with fish oil, and this result is in agreement with earlier reports showing that consumption of *n* − 3 LC-PUFA lengthens gestational duration [33]. The mechanisms of the initiation and progress of labour are poorly understood, but they are thought to involve PGE2, cytokine regulation, oxidative stress status, and the process of cell apoptosis [8, 34, 35]. Our results showed that the FD increased the activities of antioxidative enzymes but decreased the concentrations of PGE2 and proinflammatory cytokines in the plasma of sows on farrowing day, implying that the variation in gestation length might be linked to multiple factors when fish oil was included in the sow diet.

The FD increased the GSH-Px and T-SOD levels in the plasma of sows on farrowing day, which is in agreement with the results of Shen et al. in 2015 [19] and Cools et al. in 2011 [36]. Systemic oxidative stress during the late gestation period is related to the lower antioxidant nutrients and total antioxidant capability [37]. The high level of antioxidative indicators in the plasma of sows might imply a higher antioxidative capacity of sows and thus a better protection against oxidative damage. Moreover, the FD had no influence on the MDA concentrations in the plasma of sows, cord blood, and new-born piglets. This is in contrast with Cools et al. (2011), who found a linear increase of MDA and FRAP in the plasma of sows with increasing percentages of fish oil in the diet [36]. It is unclear what the reasons for these discrepancies are, but they might be related to different experimental protocols used, particularly those involving the dietary EPA level [11], the dietary storage (vacuum dark bag storage or common bag storage), and the dietary antioxidant supply [38].

The FD had no effect on MDA, GSH-Px, and T-SOD in the plasma of cord blood and new-born piglets and on the expression of SOD, GPx, and catalase (CAT) mRNA in the livers of new-born piglets. Similarly, Sarkadi-Nagy et al. in 2003 [39] and Tanghe et al. in 2013 [40] reported that the offspring did not reveal signs of an altered oxidative status when *n* − 3 PUFA were included in the sow diet. Strikingly, in our study we observed that the FD decreased the expression of SOD mRNA in placentas. There is very limited information available about the antioxidant gene expression in sow placenta. However, Su et al. in 2017 demonstrated that the placental antioxidant system of sows may have an adaptive response to oxidative stress through increasing gene expression of antioxidant enzymes in sows fed oxidized corn oil, but normalized by the supplementation of antioxidants [41]. As mentioned above, no signs of an altered oxidative status in the new-born piglet might be associated with the antioxidant defence of sow placenta, which might play an adaptive role in protecting the foetus from oxidative damage.

The maternal diet with fish oil regulates the inflammatory response by suppressing the production of proinflammatory cytokines and increasing the production of anti-inflammatory cytokines [13]. Our results showed that the FD decreased the concentration of IL-6 in the plasma of sows on farrowing day, and the FD downregulated the expression of IL-1*β* and IL-6 mRNA in placentas. Similarly, studies have reported that supplementing maternal diet with EPA or DHA results in a significant reduction in the production of IL-1*β*, IL-6, and TNF-*α* by inflammatory cells in vitro or in humans [13, 42]. IL-10 is an anti-inflammatory cytokine produced by inflammatory cells, and it is known to prevent the overactivation of proinflammatory signals at the maternal-foetal interface [43]. Oliver et al. in 2012 reported that DHA specifically enhanced anti-inflammatory IL-10 secretion [13]. Kemse et al. in 2016 reported that *n* − 3 fatty acid supplementation played a key role in reducing inflammation by maintaining the level of IL-10 in the placenta during pregnancy-induced hypertension. Our present results showed that the FD increased the IL-10 levels in the maternal plasma on farrowing day and upregulated the expression of IL-10 mRNA in the placenta.

Neonatal immune response is characterized by an uncompensated proinflammatory response that can lead to inflammation-related morbidity and increased susceptibility to infection. In our study, the FD had no effect on the concentrations of the IL-6 and TNF-*α*, but decreased the concentration of IL-1*β* in the plasma of cord blood. Moreover, the FD upregulated the expression of IL-10 mRNA in the livers of new-born piglets. Our results indicated that the maternal diet with fish oil might modulate the immune response of new-born piglets by reducing the transfer of proinflammatory cytokines from sows to their piglets via cord blood and upregulating the expression of anti-inflammatory cytokines in the livers of piglets. There is no report about the cytokine in the pig cord blood, and it is still not well understood whether the maternal diet with fish oil has an effect on the neonatal immune response in humans via the cord blood. A previous study by Espiritua et al. in 2016 with the cord blood of humans and the neonatal cord blood mononuclear cells from healthy full-term infants showed that DHA treatment produced a significant concentration-dependent inhibition of TNF-*α*, IL-6, IL-1*β*, and IL-8 secretion, but not IL-10, and administration of *n* − 3 PUFA could attenuate or resolve neonatal inflammatory response [44]. However, there is a discrepant report in pregnant humans by Mozurkewich et al. in 2018, who reported that supplementation with EPA- or DHA-rich fish oils had no significant effect on cord blood cytokine concentrations, including IL-1*β*, IL-6, TNF*-α*, and IL-10 [45].

Previous attempts to explain the immunological effects of *n* − 3 PUFAs have largely focused on the ability of these fatty acids to suppress the production of the inflammatory PGE2 [46]. PGE2 is produced from the *n* − 6 PUFA arachidonic acid (AA) by the action of prostaglandin-endoperoxide synthase 2 (PTGS2; also known as cyclooxygenase-2, COX2). EPA is also a substrate for PTGS, and the metabolism product might be linked with the initiation of labour [33]. As cellular membranes contain relatively high amounts of EPA, which is the principle eicosanoid precursor and acts as a competitive inhibitor of arachidonic acid- (AA-) derived eicosanoid synthesis [47], the production of PGE2 is decreased. Previous studies have demonstrated that PGE2 induces the production of IL-6 by macrophages [48], and it inhibits ALOX5 from upregulating the production of lipoxins that are considered to act to resolve inflammation [49]. It is documented that the maternal diet with *n* − 3 PUFA increased the proportion of EPA in the placenta and the livers of piglets [22, 23]. In this study, the FD decreased the concentration of PGE2 in the plasma of sows, but it had no effect on the PGST2 mRNA expression. Moreover, the gene expression of ALOX5 in the placentas and livers of piglets were upregulated in the FD group.

The established mechanism for the anti-inflammatory effect of *n* − 3 LC-PUFA is by the inhibition of either NF-*κ*B or MAPK activation, which regulates the production of proinflammatory cytokines such as TNF-*α* and IL [50]. Oxidative stress has been proposed to be linked with the inflammatory responses and trigger the activation of NF-*κ*B or MAPK signal pathways resulting in an increase of the inflammatory process in various cell types [51]. To investigate the molecular mechanism underlying the anti-inflammatory effect of fish oil, we targeted the MAPK and NF-*κ*B pathways. Phosphorylation of TAK1 is upstream of NF-*κ*B and JNK. Oh et al. in 2010 reported that *n* − 3 PUFA might block TAB1/TAK1 binding in monocytic cells, resulting in the inhibition of the activation of the NF-*κ*B and JNK pathway [12]. In our study, the maternal diet with fish oil suppressed the expression of TAB1 mRNA and phosphorylation of JNK in placentas, but it had no effect on the expression of NF-*κ*B mRNA and phosphorylation of I*κ*B*α* in placentas. This implies that the effect of maternal diet with fish oil on anti-inflammatory response in placentas might be via inhibiting the JNK signal pathway.

In addition, PPAR*γ* is a transcription factor that acts in an anti-inflammatory manner. It can directly regulate inflammatory gene expression, but it also interferes with the activation of the prototypical proinflammatory transcription of NF-*κ*B [47]. The FD upregulated the expression of GPR120 mRNA in the livers of piglets and tended to downregulate the expression of PPAR*γ* mRNA in the livers of piglets, indicating that the maternal diet with fish oil might modulate the neonatal immune system for adapting their living environment after birth. However, there were no effects on the NF-*κ*B and MAPK signal pathways between the two groups. The possible reasons might be linked to the periparturient immunosuppression of mammals which is helpful for the survival of offspring by decreasing the immunological rejection of the foetus and passing immune cells to foetus [52].

## 5. Conclusions

In conclusion, our data demonstrated that the maternal diet with fish oil might alleviate oxidative stress in sows on farrowing day and might modulate inflammatory response in full-term placenta by inhibiting the JNK signal pathway. Moreover, the maternal diet with fish oil might modulate the immune response of new-born piglets by reducing the transfer of proinflammatory cytokines from sows to their piglets via cord blood and upregulating the expression of anti-inflammatory cytokines in the livers of piglets. Based on our findings, it is important to pursue further studies on the development of antioxidative stress and anti-inflammatory response, as well as the relevant receptors and mechanisms in mothers and their offspring.

## Figures and Tables

**Table 1:** Ingredient composition of diets (as fed basis, %).

	Gestation diet 1 (%)		Gestation diet 2 (%)	
	SD	FD	SD	FD
Corn	35.73	35.73	45.35	45.35
Barley	30.00	30.00	25.00	25.00
Soybean hull	5.00	5.00		
Soybean meal	18.50	18.50	16.00	16.00
Fermented soybean meal	4.00	4.00	4.00	4.00
Dried porcine soluble (DPS)			2.00	2.00
Soybean oil	3.00	0.50	3.50	0.70
Fish oil		2.50		2.80
Calcium hydrogen phosphate	1.30	1.30	1.40	1.40
Limestone	1.00	1.00	1.20	1.20
NaCl	0.35	0.35	0.35	0.35
L-Threonine (98% threonine)			0.03	0.03
L-Lysine HCl (98% lysine)	0.10	0.10	0.15	0.15
Minerals and vitamins premix^1^	1.00	1.00	1.00	1.00
Antioxidant^2^	0.02	0.02	0.02	0.02
Total	100	100	100	100

SD=soybean oil diet; FD=fish oil diet. ^1^The composition of this mineral and vitamin premix (Shanghai Xinnong Feed Co. Ltd., Shanghai, China) was supplied per kg of diet as follows: retinol, 8750 IU; cholecalciferol, 2835 IU; *α*-tocopherol, 84 mg; menadione, 5.96 mg; thiamine, 1.85 mg; riboflavin, 6.57 mg; niacin, 37.6 mg; D-pantothenic acid, 29.2 mg; pyridoxine, 4.2 mg; D-biotin, 0.43 mg; folic acid, 2.5 mg; vitamin B-12, 0.04 mg; choline chloride, 500 mg; Fe, 212 mg as ferrous sulfate monohydrate; Cu, 30 mg as copper sulfate pentahydrate; Mn, 30 mg as manganese sulfate monohydrate; Zn, 144 mg as zinc sulfate monohydrate; I, 1 mg as calcium iodate; and Se, 0.3 mg as sodium selenite. ^2^Antioxidant includes 66% ethoxyquin.

**Table 2:** Fatty acid composition of the diets.

	Gestation diet 1 (%)		Gestation diet 2 (%)	
	SD	FD	SD	FD
Fatty acids (g/100 g total fatty acids)				
14 : 0	0.29	3.75	0.12	3.15
16 : 0	15.39	19.16	14.35	17.87
16 : 1	n.d.	4.79	0.20	4.23
18 : 0	3.21	2.74	3.55	3.08
18 : 1	21.33	17.60	24.10	19.72
18 : 2*n* − 6	53.73	29.96	52.49	32.65
18 : 3*n* − 3	6.08	2.56	5.20	2.62
20 : 0	n.d.	1.35	n.d.	1.10
20 : 1	n.d.	n.d.	n.d.	0.73
20 : 5*n* − 3	n.d.	9.8	n.d.	8.07
22 : 5*n* − 3	n.d.	0.87	n.d.	0.79
22 : 6*n* − 3	n.d.	6.17	n.d.	5.46
*n* − 6 : *n* − 3	8.84	1.54	10.09	1.93

SD=soybean oil diet; FD=fish oil diet; n.d.=not detectable; *n* − 3=*n* − 3 polyunsaturated fatty acid; *n* − 6=*n* − 6 polyunsaturated fatty acid; *n* − 6 : *n* − 3=*n* − 6 polyunsaturated fatty acids : *n* − 3 polyunsaturated fatty acids.

**Table 3:** Pregnancy outcome and the organ indices of new-born piglets.

	SD	FD	*P* value
Gestation length	115.00 ± 0.63	116.50 ± 0.84	0.006
Placenta weight (kg)	4.12 ± 1	4.46 ± 0.88	NS
Placental: litter weight at birth (live)	0.19 ± 0.04	0.22 ± 0.06	NS
Intestinal length: BW	284.23 ± 17.13	308.38 ± 29.75	NS
Liver weight: BW	3.76 ± 0.85	3.84 ± 0.43	NS
Brain weight: BW	21.58 ± 2.86	22.03 ± 2.16	NS
Spleen weight: BW	0.88 ± 0.09	0.91 ± 0.27	NS
Kidney weight: BW	6.87 ± 1.56	5.85 ± 0.42	NS
Pancreas weight: BW	1.04 ± 0.06	0.87 ± 0.2	NS

SD=soybean oil diet; FD=fish oil diet. Values are means ± SEM, *n* = 6. ^∗^Mean values were significantly different between the SD group and the FD group (*P* < 0.05). NS, not significant.

## Data Availability

The data used to support the findings of this study are included within the article and the supplementary information file.

## Supplementary Materials

Supplementary Materials Table S1: fatty acid composition of soybean oil and fish oil in the experimental diet. This table listed the fatty acid composition of soybean oil and fish oil used for producing the gestational diet. Table S2: primers for all target genes used for real-time PCR. This table listed all the primers that were used to determine the relative expression level of target genes in the placentas and the livers of piglets.
