# Supplementary material for: The Effect of Maternal Diet with Fish Oil on Oxidative Stress and Inflammatory Response in Sow and New-Born Piglets
Source: Oxid Med Cell Longev. 2019 Jun 2;2019:6765803. doi: 10.1155/2019/6765803 (PMC7012253; doi:10.1155/2019/6765803)
Supplement: Supplementary Materials — Table S1: fatty acid composition of soybean oil and fish oil in the experimental diet. This table listed the fatty acid composition of soybean oil and fish oil used for producing the gestational diet. Table S2: primers for all target genes used for real-time PCR. This table listed all the primers that were used to determine the relative expression level of target genes in the placentas and the livers of piglets. [file 6765803.f1.docx]

## Supplementary Table 1. Fatty acid composition of soybean oil and fish oil in experimental diets

| Item | Fish oil | Soybean oil |
| --- | --- | --- |
| Fatty acids(g/100g total fatty acids) |  |  |
| 14:0 | 7.59 | n.d1 |
| 16:0 | 17.87 | 10.57 |
| 16:1 | 9.98 | n.d |
| 18:0 | 3.54 | 3.79 |
| 18:1 | 12.52 | 23.76 |
| 18:2(n-6) | 1.19 | 53.89 |
| 18:3(n-3) | 0.80 | 8.00 |
| 20:0 | 3.15 | n.d |
| 20:1 | 1.72 | n.d |
| 20:5(n-3) | 21.12 | n.d |
| 22: 1 | n.d1 | n.d |
| 22:5(n-3) | 2.28 | n.d |
| 22:6(n-3) | 14.18 | n.d |
| n-6:n-3 | 0.03 | 6.70 |

n.d=not detectable; n-3 = n-3 poly unsaturated fatty acid; n-6 = n-6 ploy unsaturated fatty acid; n-6: n-3=n-6 polyunsaturated fatty acids: n-3 polyunsaturated fatty acids.

## Supplementary Table 2. Primers used for all target genes used for real-time PCR

| Genes | Primer sequence (5’–3’) | Product size(bp) | GenBank accession | Reference |
| --- | --- | --- | --- | --- |
| *β*-actin | F: 5'-TCATCACCATCGGCAACGAG-3' | 217 | DQ845171.1 |  |
|  | R: 5'-GCCGTGATCTCCTTCTGCAT-3' |  |  |  |
| SOD | F:5'-GAGACCTGGGCAATGTGACT-3' | 189 | GU944822.1 | [[1](#_ENREF_1)] |
|  | R:5'-CTGCCCAAGTCATCTGGTTT-3' |  |  |  |
| GPx | F:5'-GCTCGGTGTATGCCTTCTCT-3' | 103 | NM_214201.1 | [[1](#_ENREF_1)] |
|  | R:5'-AGCGACGCTACGTTCTCAAT-3' |  |  |  |
| CAT | F:5'-ACTTCTGGAGCCTACGTCCT-3' | 93 | NM_214301.2 | [[1](#_ENREF_1)] |
|  | R:5'-ATCCGTTCATGTGCCTGTGT-3' |  |  |  |
| IL-1β | F:5'-TCTGCCCTGTACCCCAACTG-3' | 64 | NM214055.1 | [[2](#_ENREF_2)] |
|  | R:5'-CCAGGAAGACGGGCTTTTG-3' |  |  |  |
| IL-6 | F:5'-ATCAGGAGACCTGCTTGATG-3' | 177 | NM_214399 | [[3](#_ENREF_3)] |
|  | R:5'-TGGTGGCTTTGTCTGGATTC-3' |  |  |  |
| TNF-α | F:5'-CCAATGGCAGAGTGGGTATG-3' | 116 | NM_214022 | [[3](#_ENREF_3)] |
|  | R:5'-TGAAGAGGACCTGGGAGTAG-3' |  |  |  |
| IL-10 | F:5'-GGTTGCCAAGCCTTGTCAG-3' | 202 | NM_214041 | [[3](#_ENREF_3)] |
|  | R:5'-AGGCACTCTTCACCTCCTC-3' |  |  |  |
| TAB1 | F:5'-CAGAGTTTGCCAAGCAGACC-3' | 175 | NM_001244067.1 |  |
|  | R:5'-CTCAGCTCGCCCAGAGGATA-3' |  |  |  |
| TAK1 | F:5'-GGCTGTTCATAACGGTACTC-3' | 204 | KU504629.1 |  |
|  | R:5'-TGGCCTTCATCTGAATACTG-3' |  |  |  |
| PTGS2 | F:5'-ATGAACGGCTGTTCCAGACG-3' | 218 | NM_214321.1 |  |
|  | R:5'-AATCTGGAAGGCGTCAGGCA-3' |  |  |  |
| ALOX5 | F: 5'-GACCCCTGCACTCTGCAGTT-3' | 201 | XM_021072736.1 |  |
|  | R:5'-GGTCTGGTGGACGTGGAAGT-3' |  |  |  |
| GPR120 | F:5'-CAGATCACCAAGGCATCAAG-3' | 206 | HQ662564.1 |  |
|  | R:5'-GGCCAGATGACCAGGTTTTG-3' |  |  |  |
| TLR-4 | F:5'-TGTGCGTGTGAACACCAGAC-3' | 136 | NM_001113039 | [[3](#_ENREF_3)] |
|  | R:5'-AGGTGGCGTTCCTGAAACTC-3' |  |  |  |
| NF-κB | F:5'-TGCTGGACCCAAGGACATG-3' | 60 | AK348766.1 | [[2](#_ENREF_2)] |
|  | R:5'-CTCCCTTCTGCAACAACACGTA-3' |  |  |  |
| PPARγ | F: 5'-GTGCGATCTTAACTGTCGGA-3' | 192 | NM214379.1 |  |
|  | R:5'-AGGTCAGCAGACTCTGGGTT-3' |  |  |  |

SOD superoxide dismutase, GPx glutathione peroxidase, CAT catalase, IL-1β Interleukin-1β, IL-6 Interleukin-6, TNF-α Tumor necrosis factor α, IL-10 Interleukin-10, TAB1 TAK1 binding protein 1, TAK1 Transforming growth factor-β activated kinase 1, PTGS2 Prostaglandin-endoperoxide synthase 2, ALOX5 lipoxygenase enzyme5, GPR120 G-protein coupled receptor 120, TLR4 Toll-like receptor 4, NF-κB Nuclear factor-B, PPARγ Peroxisome proliferator activated receptor gamma

## Reference

[1] Su, G., J. Zhao, G. Luo, et al., "Effects of oil quality and antioxidant supplementation on sow performance, milk composition and oxidative status in serum and placenta," *Lipids Health Dis*, vol. 16, no.1, pp. 107. 2017.

[2] Han, F., L. Hu, Y. Xuan, et al., "Effects of high nutrient intake on the growth performance, intestinal morphology and immune function of neonatal intra-uterine growth-retarded pigs," *The British journal of nutrition*, vol. 110, no.10, pp. 1819-1827. 2013.

[3] Pasternak, J.A., V.I.A. Aiyer, G. Hamonic, et al., "Molecular and Physiological Effects on the Small Intestine of Weaner Pigs Following Feeding with Deoxynivalenol-Contaminated Feed," *Toxins (Basel)*, vol. 10, no.1. 2018.
